# The NifA-RpoN Regulon of *Mesorhizobium loti* Strain R7A and Its Symbiotic Activation by a Novel LacI/GalR-Family Regulator

**DOI:** 10.1371/journal.pone.0053762

**Published:** 2013-01-07

**Authors:** John T. Sullivan, Steven D. Brown, Clive W. Ronson

**Affiliations:** Department of Microbiology and Immunology, University of Otago, Dunedin, New Zealand; University of Florida, United States of America

## Abstract

*Mesorhizobium loti* is the microsymbiont of *Lotus* species, including the model legume *L. japonicus. M. loti* differs from other rhizobia in that it contains two copies of the key nitrogen fixation regulatory gene *nifA, nifA1* and *nifA2*, both of which are located on the symbiosis island ICE*Ml*Sym^R7A^. *M. loti* R7A also contains two *rpoN* genes, *rpoN1* located on the chromosome outside of ICE*Ml*Sym^R7A^ and *rpoN2* that is located on ICE*Ml*Sym^R7A^. The aims of the current work were to establish how *nifA* expression was activated in *M. loti* and to characterise the NifA-RpoN regulon. The *nifA2* and *rpoN2* genes were essential for nitrogen fixation whereas *nifA1* and *rpoN1* were dispensable. Expression of *nifA2* was activated, possibly in response to an inositol derivative, by a novel regulator of the LacI/GalR family encoded by the *fixV* gene located upstream of *nifA2*. Other than the well-characterized *nif/fix* genes, most NifA2-regulated genes were not required for nitrogen fixation although they were strongly expressed in nodules. The NifA-regulated *nifZ* and *fixU* genes, along with *nifQ* which was not NifA-regulated, were required in *M. loti* for a fully effective symbiosis although they are not present in some other rhizobia. The NifA-regulated gene *msi158* that encodes a porin was also required for a fully effective symbiosis. Several metabolic genes that lacked NifA-regulated promoters were strongly expressed in nodules in a NifA2-dependent manner but again mutants did not have an overt symbiotic phenotype. In summary, many genes encoded on ICE*Ml*Sym^R7A^ were strongly expressed in nodules but not free-living rhizobia, but were not essential for symbiotic nitrogen fixation. It seems likely that some of these genes have functional homologues elsewhere in the genome and that bacteroid metabolism may be sufficiently plastic to adapt to loss of certain enzymatic functions.

## Introduction

*Mesorhizobium loti* is the natural microsymbiont of *Lotus* species, including the model legume *L. japonicus*. The genes required for nodule formation and nitrogen fixation in *M. loti* strain R7A are located on a 502-kb chromosomally located symbiosis island [1], [2], which was subsequently named ICE*Ml*Sym^R7A^ [3] as it belongs to the family of mobile genetic elements collectively termed integrative and conjugative elements (ICEs) [4]. Sequence analysis of ICE*Ml*Sym^R7A^ revealed that it shares 248 kb of DNA with the 611-kb symbiosis island of the sequenced *M. loti* strain MAFF303099 [5], including all the genes likely to be required for Nod factor synthesis and the formation of a functional nitrogenase enzyme. In addition, it contains mobility genes, a type IV secretion system similar to that of the *vir* system from *Agrobacterium tumefaciens* and a diverse range of regulators, metabolic genes, and transporters that may contribute to nodule function [6], [7].

Compared to several other rhizobial species, very little is known about how *M. loti* genes required for symbiotic nitrogen fixation are regulated. In most nitrogen-fixing bacteria, the NifA protein binds to an upstream activating sequence (UAS) and acts in association with the RNA polymerase sigma factor RpoN (σ54) to activate *nif* gene expression and, in rhizobia, the expression of several other symbiotic genes (reviewed in [8]). *M. loti* differs from other rhizobia examined to date in that it contains two copies of the *nifA* gene, *nifA1* and *nifA2*, both of which are located on ICE*Ml*Sym^R7A^. The *nifA1* gene is most similar to and in the same genomic context (between *fixX* and *nifB*) as *nifA* from *Rhizobium etli, R. leguminosarum, Rhizobium* sp. strain NGR234 and *Sinorhizobium meliloti* [9], [10], [11], [12], [13]. In contrast, *nifA2* is most similar to *nifA* from *Bradyrhizobium japonicum* and is not located adjacent to known nitrogen fixation genes. The two genes are not functionally redundant as *M. loti nifA2* mutants form Fix^−^ nodules [14], [15] whereas *nifA1* mutants are not symbiotically impaired [14].

NifA activity in rhizobia is oxygen-sensitive and it is thought that conserved cysteine residues present within NifA are involved in sensing and reacting to the cellular oxygen status (reviewed in [8]). In addition, in most rhizobia the *nifA* gene is subject to transcriptional regulation although the mechanisms vary depending on the rhizobial strain. In *S. meliloti nifA* expression is activated by the FixLJ two-component regulatory system in response to low oxygen tension. In addition *nifA* is located downstream of *fixABCX* and *nifA* expression is enhanced by NifA-mediated expression via the *fixA* promoter [8]. In *B. japonicum* the *fixR-nifA* operon is controlled by the redox-responsive two-component system RegSR acting on the *fixR*p1 promoter [16]. In *R. leguminosarum* bv. *viciae* strain UPM791, *nifA* is expressed only under symbiotic conditions, through autoregulation via the promoter which precedes the *orf71-orf79-fixW-orf5-fixABCX-nifA* operon. Basal symbiotic expression of *nifA* occurs from an unidentified promoter upstream of the 3′-end of *fixX* [17]. For *R. etli*, expression of *nifA* occurs independently of cellular oxygen status and no genetic regulatory elements have been identified. However *nifA* expression is upregulated under symbiotic conditions, suggesting that it may be under some form of symbiosis-specific regulation [18].

RpoN recognizes and binds a −24/−12 promoter sequence with the consensus 5′-TG**G**CACG-N4-TTG**C**W-3′. The G situated at position −24 and C situated at −12 relative to the transcription start site (shown in bold in consensus) are almost invariant although the *nifH* promoters of *M. loti* and *R. etli* have an A instead of C at the −12 position. Sixteen candidate NifA-regulated promoters were defined on ICE*Ml*Sym^R7A^ on the basis of their containing a potential NifA upstream activator sequence (TGT-N10-AGA) and a −24/−12 promoter sequence [6]. Of these, 15 are located upstream of annotated genes, including eight that precede known *nif/fix* gene clusters (Table 1). One potential promoter region was found upstream of *msi281* but in reverse orientation, facing a 2.3-kb region that contains a fragment of the nodulation gene *noeL* but no annotated complete genes. The *msi320-msi321* cluster is the only potential NifA-regulated cluster present on ICE*Ml*Sym^R7A^ that is not present in MAFF303099 [6]. Whether the *M. loti* genes in the putative NifA-regulated clusters other than the well-characterised *nif/fix* genes are required for symbiotic nitrogen fixation remains unknown. However many of them have predicted functions that may be of symbiotic relevance (Table 1).

**Table 1:** Potential NifA-regulated operons on ICE*Ml*Sym^R7A^.

Gene or operon	−24/−12 promoter seq.	Putative gene/operon function
*omp2b* (*msi036*)	TTGGCACGTCATTTGCG	Outer-membrane porin (Omp2 family)
*msi071-msi064*	TTGGCACGAGTTTTGAA	Diterpenoid synthesis
*msi158*	TTGGCACGACACATGCG	Outer-membrane porin (OmpW family)
*msi262-msi263*	CTGGCACGTTCTGTGCA	Msi262 iron-sulfur cluster assembly, HesB family, IscN; Msi263 FeS cluster assembly, NifU N-terminal homology
*acdS* (*msi273*)	TTGGCACGGTACATGCT	1-aminocyclopropane-1-carboxylate deaminase
*fixV* frag, *hypC* frag, *msi276-274*	CTGGCATGACGTTTGCT	Msi276 DUF683 (found in *nif* clusters); Msi275 FdxB Ferredoxin III [4Fe-4S], *nif*-specific; Msi274 partial similarity (SyrA superfamily)
*msi280*	CTGGCACGTTCGATGCA	L-lysine 6-monooxygenase
Nr *msi281*	CTGGCACGGCCTTTGCT	no annotated genes
*nifHDKENX-msi288*	TTGGCACGAGTTTTGAA	Nitrogenase enzyme synthesis; msi288 unknown function DUF269, NifX-associated protein
*nifH* frag, *msi321-320*	TTGGCACGAGTTTTGAA	Msi321 methyltransferase; Msi320 unknown
*nifQ* frag, *msi332-331*	TTGGCACGACTTTTGAA	Msi332 cytochrome P450 monooxygenase; Msi331 DUF1271 superfamily, possible ferredoxin
*prxS-rpoN2* (*msi334-msi335*)	ATGGCACGGCGCTTGCG	peroxiredoxin; Sigma 54 subunit of RNA polymerase
*nifS-nifW* (*msi340-341*)	TTGGCACGGTCCATGCG	NifS cysteine desulfurase, iron-sulfur cluster synthesis; NifW nitrogenase-stabilizing/protective protein
*fixABCX-nifA1* (*msi342-346*)	TTGGCACGAATGATGCT	Electron transport to nitrogenase; Nif-regulatory protein
*nifB-fdxN-nifN-fixU-msi351* (*msi347-351*)	TTGGCATATCTCTTGCG	Nitrogenase synthesis; Msi351 conserved hypothetical, prokaryotic sirtuin-like family
*ccpR* (*msi380*)	TTGGCACGACTTTTGAT	Cytochrome c peroxidase

*M. loti* also contains two *rpoN* genes, *rpoN1* (*mll3196* in strain MAFF303099) located on the chromosome outside of the symbiosis island and *rpoN2* that is located on the island (*msi335* in strain R7A). *R. etli* also contains *rpoN1* and *rpoN2* genes and RpoN1 is required for the metabolism of C4-dicarboxylic acids and several nitrogen sources during free-living growth [19] while RpoN2 is involved in symbiotic nitrogen fixation. The *rpoN2* gene is part of a *prxS-rpoN2* operon that is activated by NifA. An additional symbiosis-specific weak promoter is located between *prxS* and *rpoN2* [20], [21]. In *M. loti* the *rpoN2* gene is also downstream of *prxS* as part of a potential NifA-regulated operon (Table 1). *B. japonicum* also has two *rpoN* genes but both RpoN1 and RpoN2 are functional in free-living and symbiotic conditions [22]. In contrast, *S. meliloti* and *R. leguminosarum* bv. *viciae* strain VF39SM have a single copy of *rpoN* that in *R. leguminosarum* is negatively autoregulated [23], [24].

A transcriptome macroarray analysis based on the *M. loti* MAFF303099 genome revealed clusters of genes within the symbiosis island that were up-regulated during symbiosis compared to free-living growth, whereas genes outside the island were in general down-regulated. The up-regulated genes included island genes involved in metabolism as well as *nif-fix* genes and the duplicate *fixNOQP* genes outside the symbiosis island [25].

The aims of the current work were to characterise the NifA-RpoN regulon in *M. loti* and to establish how *nifA* expression is activated. In addition we wished to determine the symbiotic phenotypes of selected metabolic genes found to be up-regulated in nodules and to determine whether their expression depended on NifA. We show that symbiotic gene expression in *M. loti* is under novel regulation and identify several new symbiotic genes. The availability of the *M. loti* mutants described in this paper should assist future studies of the physiological functioning of nodules formed on the model legume *Lotus japonicus*.

## Results

### NifA2 but not NifA1 is required for symbiotic nitrogen fixation although *nifA1* encodes a functional protein

Previous work showed that *M. loti nifA2* mutants form Fix^−^ nodules [14], [15] whereas *nifA1* mutants are not symbiotically impaired [14]. For the current work, marker exchange deletion mutants JS01 (Δ*nifA1*:: Ωkan) and JS02 (Δ*nifA2*:: Ωkan) were constructed. As expected, JS01 formed Fix^+^ nodules on *Lotus corniculatus* whereas JS02 was Fix^−^ (Table 2). Plasmid pJS100 that contained *nifA2* and the preceding 626 bp cloned into vector pFAJ1700 (Table 3) complemented strain JS02 to Fix^+^, confirming that the Fix^−^ phenotype of the *nifA2* mutation was not due to a polar effect on downstream genes.

**Table 2:** *M. loti* mutants constructed in this study and their symbiotic phenotypes.

Strain Background			Description^a^	R7A mutant Fix phenotype^b^
R7A	JS01	JS02		
JS01			Δ*nifA1*:: Ω*kan*, gene replacement deletion	+
JS02			Δ*nifA2*:: Ω*kan*, gene replacement deletion	−
JS03		JS229	*nifA1*::*lacZ*, pFUS2 IDM	+
JS04	JS121	JS221	*rpoN1*::*lacZ*, pFUS2 IDM	+
JS05A	JS114	JS214	Δ*rpoN2*:: Ω*kan*, gene replacement deletion	−
JS05B			*rpoN2*::*lacZ*, pFUS2 IDM	−
JS06A	JS112	JS212	*prxS*::*lacZ*, pFUS2 IDM	−
JS06B	JS113	JS213	Δ*prxS*, markerless in-frame deletion	+
JS07			*prxS*::*lacZ*, pFUS2 CMD	+
JS08			*fixK*::*lacZ*, pFUS2 IDM	+
JS09			*fixJ*::*lacZ*, pFUS2 IDM	+
JS10			*regR*::*lacZ*, pFUS2 IDM	+
JS11			*regS*::*lacZ*, pFUS2 IDM	+
JS12			Δ*regR*, markerless deletion mutant	+
JS13			JS12 *fixK*::*lacZ*, pFUS2 IDM	+
JS14			JS12 *fixJ*::*lacZ*, pFUS2 IDM	+
JS15	JS119		*nifA2*::*lacZ*, pFUS2 CMD, 536 bp of *nifA2* promoter preceding *lacZ*	+
JS16			*nifA2*::*lacZ*, pFUS2 CMD, 426 bp of *nifA2* promoter preceding *lacZ*	−
JS17			*nifA2*::*lacZ*, pFUS2 CMD, 293 bp of *nifA2* promoter preceding *lacZ*	−
SB01			Δ*fixV*:: Ω*kan,* gene replacement deletion of *msi360*, renamed *fixV* in this work	−
JS18			*fixV*::*lacZ*, pFUS2 IDM	−
JS19			SB01 *nifA2*::*lacZ*, pFUS2 CMD	−
JS20			SB01 *fixA*::*lacZ*, pFUS2 CMD	−
JS21	JS111	JS211	*nifH*::*lacZ*, pFUS2 IDM	−
JS22	JS116	JS216	*nifS*::*lacZ*, pFUS2 IDM	−
JS23	JS118	JS218	*nifB*::*lacZ*, pFUS2 IDM	−
JS24	JS103	JS203	*msi158*::*lacZ*, pFUS2 IDM	P
JS25	JS101	JS201	*msi036*::*lacZ*, pFUS2 IDM	+
JS26			Δ*msi036*:: Ω*kan msi158::lacZ*, gene replacement deletion of *msi036*, pFUS2 IDM of *msi158*	P
JS27			Δ *[msi262-msi263]*:: Ω*kan,* gene replacement deletion of *msi262-msi263*	P
JS28			Δ *[fdxN-fixU]*:: Ω*kan*, gene replacement deletion of *fdxN-nifZ-fixU*	−
JS29			Δ *[nifZ-fixU]:* Ω*kan*, gene replacement	−
JS30			Δ*fixU*:: Ω*kan*, gene replacement deletion	+
JS31			*msi351*::*lacZ*, pFUS2 IDM	+
JS32	JS120	JS220	*ccpR*::*lacZ*, pFUS2 IDM	+
JS33			JS07 *ccpR*::*lacZ*, pFUS2 IDM	+
JS34	JS102	JS202	*msi071*::*lacZ*, pFUS2 IDM	+
JS35	JS124	JS224	*msi083*::*lacZ*, pFUS2 IDM	+
JS36	JS123	JS223	*metE*::*lacZ*, pFUS2 IDM	+
JS37	JS122	JS222	*metK*::*lacZ*, pFUS2 IDM	+
JS38	JS126	JS226	*pepM*::*lacZ*, pFUS2 IDM	+
JS39	JS104	JS204	*msi260*::*lacZ*, pFUS2 IDM	+
JS40	JS105	JS205	*msi262*::*lacZ*, pFUS2 CMD	P
JS41	JS106	JS206	*acdS*::*lacZ*, pFUS2 IDM	+
JS42	JS128	JS228	*aatA*::*lacZ*, pFUS2 IDM	+
JS43	JS127	JS227	*asnB*::*lacZ,* pFUS2 IDM	+
JS44	JS125	JS225	*exsA*::*lacZ*, pFUS2 IDM	+
JS45	JS115	JS215	*nifQ*::*lacZ,* pFUS2 IDM	P
JS46			*msi338*::*lacZ*, pFUS2 CMD	+
JS47			Δ*msi337*:: Ω*kan*, gene replacement deletion	P
JS48			Δ*msi338*:: Ω*kan*, gene replacement deletion	P
JS49	JS108	JS208	*msi280*::*lacZ*, pFUS2 IDM	+
JS50			Δ *[msi274-276]*:: Ω*kan*, gene replacement deletion of *msi274-msi275-msi276*	+
JS51	JS107	JS207	*msi276*::*lacZ*, pFUS2 CMD	+
JS52	JS109	JS209	*msi321*::*lacZ*, pFUS2 IDM	+
JS53	JS110	JS210	*msi332*::*lacZ*, pFUS2 CMD	+
JS54	JS117	JS217	*fixA*::*lacZ*, pFUS2 CMD	+

a IDM  =  insertion duplication mutant in which coding sequence disrupted; CMD  =  *cis*-merodiploid insertion mutant in which gene is not inactivated as mutant retains wild-type copy of gene including entire promoter region downstream of *lacZ* fusion (except for JS16 and JS17 in which promoter is truncated).

b Symbiotic effectiveness of mutants determined by measuring the wet weights of 15 *L. corniculatus* seedlings at 6 weeks post-inoculation. Data were compared with those obtained for seedlings inoculated with the wild-type and uninoculated controls. +  =  Fix^+^ (fully effective); −  =  Fix^−^ (ineffective); P  =  partially effective (see Table 5).

**Table 3:** Plasmids used in this study.

Plasmid	Description	Reference
pFAJ1700	Broad-host-range IncP plasmid, Tc^R^	[69]
pFAJ1708	pFAJ1700 containing *nptII* promoter	[69]
pFUS2	*oriC* ^ColE1^ *oriT* ^RK2^ *lacZ* transcriptional reporter; suicide vector, Gm^R^	[62]
pIJ3200	Broad-host-range IncP plasmid, Tc^R^	[70]
pPH1JI	IncP plasmid, Gm^R^	[71]
pJQ200SK	Suicide vector containing *sacB* gene, Gm^R^	[65]
pJS100	pFAJ1700 containing *nifA2* and preceding 626 bp	This study
pJS101	pFAJ1700 containing the 626 bp that precedes *nifA2* fused at the start codon to the complete *nifA1* gene	This study
pJS102	pFAJ1700 containing *rpoN1* and preceding 118 bp	This study
pJS103	pFAJ1700 containing the 570 bp that precedes *prxS* fused at the start codon to the complete *rpoN2* gene	This study
pJS104	pFAJ1700 containing *fixV* and preceding 295 bp	This study
pJS105	pFAJ1700 containing *msi158* and preceding 392 bp	This study
pJS106	pFAJ1700 containing *msi262* and preceding 739 bp	This study
pJS107	pFAJ1700 containing 279 bp upstream of *nifB*, with in-frame deletion of *nifB* and complete *fdxN, nifZ* and *fixU* genes	This study
pJS108	pFAJ1700 containing the *nifB* promoter region, with the 5′ end of *nifB* fused in-frame to the 3′ end of *fdxN*, and complete *nifZ* and *fixU* genes.	This study
pJS109	pFAJ1700 containing the *nifB* promoter region, with the 5′ end of *nifB* fused in-frame to the 3′ end of *nifZ*, and complete *fixU* gene.	This study
pJS110	pFAJ1700 containing *nifQ* cloned behind *nptII* promoter	This study

To determine whether *nifA1* was expressed in nodules, an insertion-duplication (IDM) mutant with a transcriptional fusion between the 5′-end of the mutated gene and *lacZ* was constructed by integration through homologous recombination of the suicide vector pFUS2 containing a cloned internal fragment of the gene. Examination of expression of the *lacZ* fusion in JS03 (*nifA1*::*lacZ*) revealed that *nifA1* was expressed. However the same fusion was not expressed in a *nifA2* mutant strain (Table 4), suggesting that *nifA1* transcription initiated from the *fixA* promoter and not the region immediately upstream of *nifA1*. To determine if *nifA1* encoded a functional protein, the region upstream of *nifA2* spanning from the 3′ end of *msi360*, the gene that precedes *nifA2*, to the *nifA2* start codon was joined to the *nifA1* gene at the start codon by extension overlap PCR. The product was cloned into pFAJ1700 and the resulting plasmid pJS101 complemented the *nifA2* mutant JS02 to a fully Fix^+^ phenotype. Taken together these results indicate that *nifA1* is functional in nodules formed by R7A, but its expression is dependent on NifA2.

**Table 4:** Symbiotic expression of various genes in wild-type, Δ*nifA1* and Δ*nifA2* backgrounds.

Gene fusion	a ß-galactosidase activity (Miller units) in:		
	R7A	JS01 (Δ*nifA1*)	JS02 (Δ*nifA2*)
No *lacZ* fusion	6.4±1.5	5.0±0.7	10.0±3.2
***nifA*** ** genes**			
*nifA1*::*lacZ*	297.9±191.2	ND	10.6±1.6*
*nifA2*CMD::*lacZ*	307.5±96.3	293.1±134.2	ND
**Genes with NifA/RpoN promoters**			
*acdS::lacZ*	369.4±167.7	547.2±111.9	7.5±4.4*^+^
*ccpR*::*lacZ*	347.7±70.6	268.8±73.3	7.6±1.8**^++^
*fixA*CMD::*lacZ*	527.8±145.4	355.5±74.8	7.8±2.4**^+^
*msi036*::*lacZ*	275.8±39.9	76.5±16.6^••^	4.3±1.6*^+^
*msi071*::*lacZ*	618.4±59.6	646.0±157.9	6.3±1.2*^+^
*msi158*::*lacZ*	1281.7±251.8	1150.0±211.1	5.5±0.9**^++^
*msi260*::*lacZ*	1339.3±72.5	685.9±374.5^•^	5.1±2.1**^+^
*msi262*CMD::*lacZ*	613.2±120.7	424.5±139.3	7.4±1.3*^+^
*msi276*CMD::*lacZ*	552.9±108.0	301.7±106.8	14.2±7.8*^+^
*msi280*::*lacZ*	371.6±125.7	268.9±101.4	7.3±1.3*^+^
*msi321*::*lacZ*	319.8±84.7	205.4±63.8	11.7±2.1*^+^
*msi332*::*lacZ*	446.6±71.4	273.5±89.9	14.6±10.2**^+^
*nifB*::*lacZ*	684.9±79.7	535.7±89.8	28.6±23.8**^+^
*nifH*::*lacZ*	854.8±126.1	610.6±86.6	9.4±7.2**^++^
*nifS*::*lacZ*	492.5±144.3	413.0±125.7	5.9±3.7*^+^
*prxS*::*lacZ*	204.1±31.1	188.8±36.1	17.5±12.9*^+^
*prxS*CMD::*lacZ*	779.8±183.1	707.3±174.8	6.1±3.4*^+^
*rpoN2*::*lacZ*	138.7±50.8	118.0±25.1	19.4±2.0*^+^
**Genes without NifA/RpoN promoters**			
*aatA*::*lacZ*	2455.8±110.6	2289.8±314.0	16.2±7.4**^++^
*asnB*::*lacZ*	861.7±107.8	809.2±174.9	22.0±6.7**^+^
*exsA*::*lacZ*	495.5±223.2	524.0±166.5	8.7±0.7*^+^
*metE*::*lacZ*	223.6±91.9	171.5±43.4	28.1±17.3*^+^
*metK*::*lacZ*	730.2±227.2	467.1±157.2	11.1±7.3**^+^
*msi083*::*lacZ*	280.0±85.4	259.6±117.0	217.8±98.9
*nifQ*::*lacZ*	200.6±112.2	149.3±108.3	9.7±10.0*^+^
*pepM*::*lacZ*	827.1±107.4	625.1±168.8	7.7±2.0**^+^
*rpoN1*::*lacZ*	29.7±6.0	25.1±7.2	24.9±8.5

a ß-galactosidase assays were performed on bacteroid suspensions from nodules harvested 14 days post-inoculation. All activity values are the average of at least two assays ± Standard Deviation. Significant differences in expression observed between R7A and R7AΔ*nifA1*:: Ωkan **  = *P*<0.005, between R7A and R7AΔ*nifA2*:: Ωkan ^++^ =  *P*<0.005, between R7AΔ*nifA2*:: Ωkan and R7AΔ*nifA1*:: Ωkan^•^ = *P*<0.05 (as determined by unpaired t test).

### RpoN2 but not RpoN1 is required for symbiotic nitrogen fixation

To determine the roles of the two *M. loti* genes that each encode the sigma factor RpoN, an IDM mutant of *rpoN1* (strain JS04) and marker exchange deletion (R7AΔ*rpoN2*:: Ω*kan*, strain JS05A) and IDM (*rpoN2*::*lacZ*, strain JS05B) mutants of *rpoN2* were constructed in the R7A background. When plated on RDM agar containing succinate as carbon source, growth of the *rpoN1* mutant was severely reduced whereas the *rpoN2* mutants grew at the wild-type rate. The *rpoN1* mutant formed microcolonies on the plates after 7 days, presumably as a result of scavenging carbon sources present in the agar. Growth was restored by plasmid pJS102 that contains the *rpoN1* gene and the preceding 118 bp cloned into pFAJ1700. When assayed on *L. corniculatus*, the *rpoN1* mutant formed Fix^+^ nodules whereas the *rpoN2* mutants were Fix^−^. These results suggested that *rpoN2* was not expressed in free-living *M. loti* but was essential for symbiotic nitrogen fixation.

The *prxS* gene that encodes an atypical 2-Cys peroxiredoxin is located immediately upstream of the *rpoN2* gene and is preceded by a potential NifA-regulated promoter (Table 1). A *prxS* IDM mutant JS06A formed Fix^−^ nodules. To ascertain if the Fix^−^ phenotype of the *prxS* mutation was due to a polar effect on *rpoN*2 expression, a *prxS* markerless in-frame deletion mutant JS06B was constructed. This strain formed Fix^+^ nodules. The 570-bp region preceding the *prxS* start codon was then amplified by PCR and fused to the *rpoN2* gene to give plasmid pJS103. This plasmid complemented both mutant strains JS06A and JS06B to a Fix^+^ phenotype, confirming that *prxS* was not required for an effective symbiosis and that *rpoN2* was expressed from the *prxS* promoter.

### FixLJK and RegSR are not required for symbiotic nitrogen fixation

In order to determine if genes known to mediate *nifA* expression in other rhizobia were involved in regulating *nifA2* in *M. loti*, IDM mutants were constructed for the R7A *fixK* (*mll6606* in MAFF303099), *fixJ* (*mll6578*), *regR* (*mlr5308*), and *regS* (*mlr5307*) genes. The resulting mutants, strains JS08 to JS11, all formed Fix^+^ nodules. Double mutants JS13 (Δ*regR fixK*::*lacZ*) and JS14 (Δ*regR fixJ*::*lacZ*) mutants were then constructed and also formed Fix^+^ nodules. Southern hybridization analysis carried out to confirm the mutants suggested that only a single copy of each of these genes was present in the R7A genome, as is the case for MAFF303099 [5].

Taken together, the above results indicate that the regulation of *nifA* expression in *M. loti* differs from that established for other rhizobial species examined to date. The results are most similar for those found with *R. etli* where no regulators of *nifA* expression have yet been found.

### *nifA2* expression is not autoregulatory

**Figure 1:**
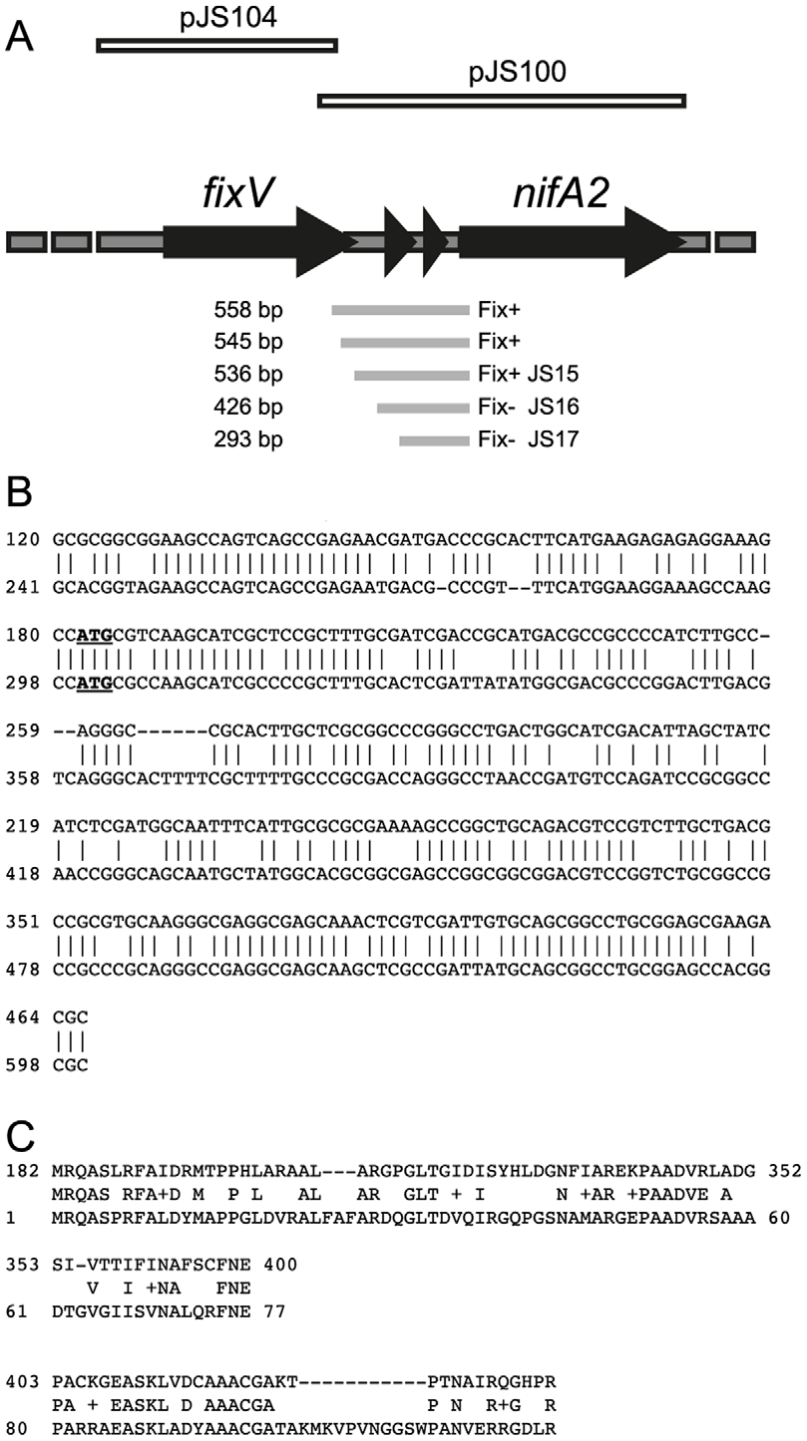
*The fixV-nifA2* region. A. Map of the *fixV-nifA2* region. The location of gene fragments in the intergenic region with homology at the protein level to Msi109 is shown. The inserts in the plasmids pJS104 and pJS100 used for complementation of *fixV* and *nifA2* mutants respectively are indicated above the map. Below the map are the intergenic fragments used to locate the *nifA2* promoter. The sizes of the intergenic fragments are shown on the left and the Fix phenotype of the resultant strains on the right. B. BlastN output showing nucleotide identity between the *fixV-nifA2* intergenic region and the *msi109* region. The ATG corresponding to the start codon of *msi109* is bolded and underlined. C. BlastX output showing amino-acid similarity between the two fragments in the *fixV-nifA2* intergenic region and Msi109.

The intergenic region (ICE*Ml*Sym^R7A^ coordinates 436876–437433) between *msi360*, the gene upstream of *nifA2*, and *nifA2* comprises 558 bp (Fig. 1A). BlastN [26] searches carried out using this region as a query showed that it shared 70% nucleotide identity from bp 120–466 with another region conserved between the R7A and MAFF303099 symbiosis islands (Fig. 1B). BlastX analysis showed the presence of two gene fragments spanning bp 182–465 (ICE*Ml*Sym^R7A^ coordinates 437058–437341) sharing highest similarity (approximately 45% amino-acid identity) with the N-terminal end of the *msi119* gene product that encodes a putative sugar epimerase (COG4130) (Fig. 1A, C).

To delineate the *nifA2* promoter region, a series of *nifA2-lacZ* nested promoter fusion strains were constructed using the suicide vector pFUS2. The pFUS2 clones were constructed using a series of nested PCR products amplified using a primer nifA2CMDR located within the 5′ end of *nifA2* and a series of 5 primers (*nifA2*CMDL1-5) located at intervals between the 3′ end of *msi360* and the 5′ end of *nifA2* (Fig. 1A). Insertion of the plasmids into the genome created five *cis*-merodiploid (CMD) strains. In these strains the full intergenic region along with the 5′-end of the *nifA2* gene was fused to *lacZ* while the amplified promoter region was fused to an intact copy of *nifA2* downstream of the inserted plasmid.

The shortest clone that gave a Fix^+^ phenotype was JS15 that contained a 536-bp region preceding the *nifA2* start codon, whereas strains JS16 and JS17 that contained 426-bp and 293-bp regions preceding the start codon respectively were Fix^−^ (Fig. 1A). This indicated that the *nifA2* promoter was located upstream of the gene fragments homologous to *msi119* located in the *msi360-nifA2* intergenic region. ß-galactosidase assays carried out on bacteroids extracted from nodules of plants 14 days post-inoculation with strains JS15, JS16 and JS17 revealed no significant differences in expression measured from the intact *nifA2* promoter-*lacZ* fusion in the strains. The Fix^+^ strain JS15 that contains the full-length promoter in front of both the *nifA2* gene and the *nifA2*::*lacZ* fusion gave 307.5±55.6 Miller Units. In comparison, the Fix^−^ strains JS16 and JS17 that contain the same *nifA2*::*lacZ* fusion but an inactive *nifA2* gene gave 304.8±63.2 and 337.2±103.9 Miller Units, respectively. These data showed that *nifA2* was not autoregulated, consistent with the absence of NifA and RpoN binding sites within the putative *nifA2* promoter region.

### A novel regulatory protein FixV activates *nifA2* expression

**Figure 2:**
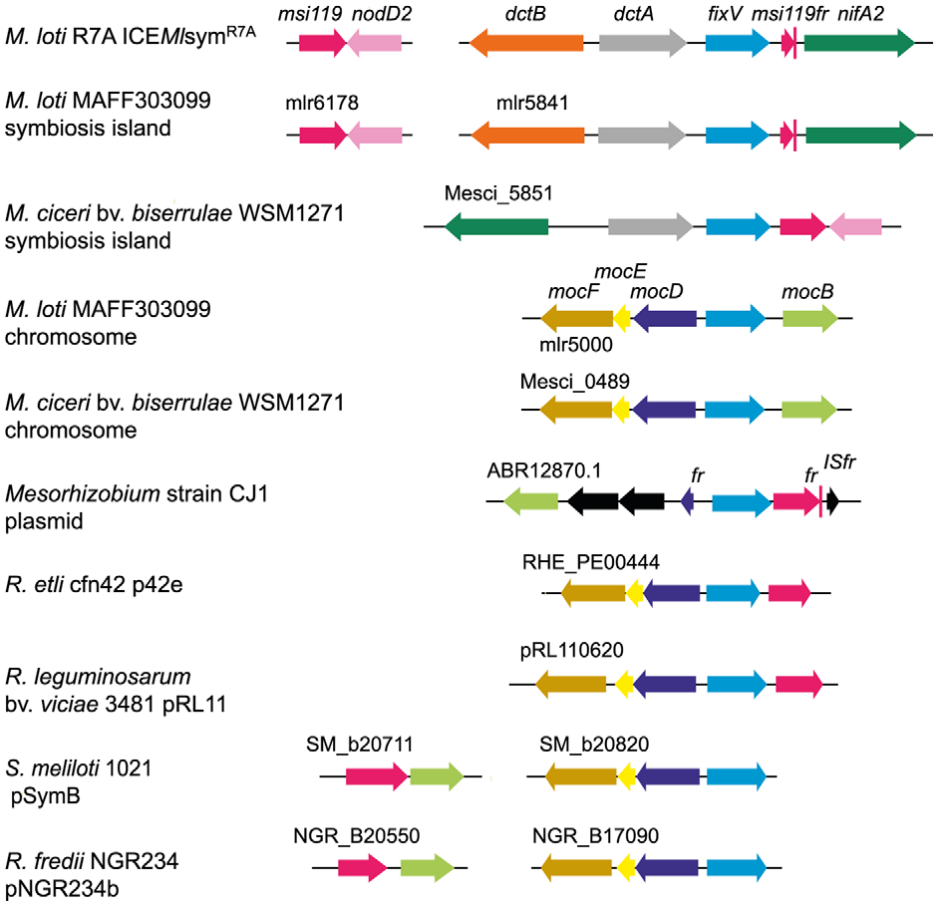
Comparison of the genetic organization of gene clusters associated with *fixV* homologs in a range of rhizobial species. Genes are shown as arrow symbols and are to scale; colours specific for each gene are used to indicate genes that encode similar proteins in other clusters. Black indicates genes lacking homology to any other genes within the clusters. Fr notes gene fragment, IS denotes insertion sequence.

*nifA2* is preceded by *msi360* which encodes a regulator of the LacI/GalR family. BLAST searches revealed that the most closely related Msi360 orthologs (approx. 60–76% amino-acid identity) are found within other rhizobial species including *Mesorhizobium ciceri, R. etli, R. leguminosarum* and non-symbiotic *Mesorhizobium* strain CJ1. In many cases the genes encoding these regulators preceded genes encoding sugar epimerases homologous to Msi119. In several rhizobia, the *msi360* homolog was also divergently transcribed from the *mocD* operon required for catabolism of the rhizopine L-3-O-methyl-scyllo-inosamine (Fig. 2). Mutants in *msi360* were constructed by marker replacement (strain SB01; Δ*msi360*:: Ωkan) and insertion duplication (strain JS18; *msi360*::*lacZ*). The mutants were symbiotically defective and wet weights of inoculated plants were not significantly different from uninoculated controls.

Several lines of evidence indicated that the *msi360* mutations were not polar on *nifA2* expression. Both SB01 and JS18 were complemented to a fully Fix^+^ phenotype by plasmid pJS104. This plasmid contains a PCR product containing *msi360* and the preceding 295 bp (Fig. 1A). Furthermore strain JS15 which contains pFUS2 inserted between *fixV* and *nifA2* was Fix^+^. Finally the *nifA2*-complementing plasmid pJS100 (Fig. 1A) failed to complement JS18 to Fix^+^.

To examine *nifA2* expression in the *msi360* mutant background, strain JS19 (Δ*msi360*:: Ωkan *nifA2*::*lacZ*-CMD) was constructed. Expression of *nifA2* in 2-week-old nodules formed by this strain was largely abolished (15.1±6.6 Miller Units compared to 307.5±55.6 Miller Units for strain JS15 (*nifA2*::*lacZ*-CMD)), indicating that Msi360 either directly or indirectly activates *nifA2* expression. We also introduced a *fixA*::*lacZ*-CMD fusion into SB01, creating JS20 (Δ*msi360*:: Ωkan *fixA*::*lacZ*-CMD). Analysis of *lacZ* expression in bacteroids from nodules formed by this strain showed that *fixA* expression was abolished in the *msi360* mutant background (9.6±7.6 Miller Units compared to 527.8±145.4 Miller Units for strain JS54 (R7A *fixA*::*lacZ-*CMD), consistent with a lack of *nifA2* expression. These results led us to rename *msi360* as *fixV* in recognition of its contribution to the regulation of *nifA2* expression.

### Expression analysis of genes preceded by NifA-regulated promoters

Expression studies were carried out to confirm that the 15 putative *nifA*-regulated promoters present on ICE*Ml*Sym^R7A^ that preceded intact genes were subject to NifA-mediated regulation, and to determine whether NifA1 influenced expression from any of these promoters. In most cases, IDM mutants of the first gene downstream of each promoter were constructed using the suicide vector pFUS2 in the wild-type (R7A), Δ*nifA1* (JS01) and Δ*nifA2* (JS02) strain backgrounds, although in a few cases CMD recombinant strains that did not inactivate the gene were also constructed. The resultant strains contained transcriptional fusions of the mutated gene to *lacZ*, enabling both the symbiotic phenotype and the expression of the gene to be determined. Mutants in the JS01 background were designated JS101 through to JS128, and those in the JS02 background JS201 through to JS228 (Table 2).

All putative NifA-regulated genes examined were strongly expressed in bacteroids harvested from nodules at two weeks post-inoculation in both the R7A and JS01 backgrounds and expression was abolished in the JS02 background, indicating *nifA2* was an absolute requirement for their expression under symbiotic conditions (Table 4). In most cases, expression of the fusion in the JS01 background was less than in the R7A background, but with the exception of *msi036*::*lacZ,* the differences were not statistically significant.

### Symbiotic phenotypes of NifA-regulated genes

All IDM and CMD mutant strains were assessed for nitrogen-fixing ability on *L. corniculatus* to determine whether the mutated gene had a symbiotic role, and to confirm that the CMD strains remained Fix^+^. Visual observations of plant growth and colour were made and wet weights were measured at six weeks post-inoculation. The fixation phenotypes of all recombinant strains are summarised in Table 2. Only strains JS02 JS05A, JS05B, JS06A, JS06B, JS21, JS22, JS23, JS28, and JS29 containing mutations within *nifA2, rpoN2, prxS, nifH, nifS, nifB, fdxN* and *nifZ* respectively were completely Fix^−^ (Table 2), producing nodulated seedlings that were otherwise indistinguishable from uninoculated seedlings that displayed severe signs of nitrogen deficiency. However a strain carrying a mutation in *msi158* (JS24), a marker exchange mutant in which *msi262* and *msi263* were deleted (JS27), and JS30, a marker exchange *fixU* mutant, were partially effective, as plants inoculated with these strains showed growth intermediate between fully Fix^+^ and Fix^−^ (Tables 2 and 5). The other mutants and all CMD strains tested (with the exception of the *nifA2*::pFUS2 CMD strains JS16 and JS17, see above) formed fully effective nodules (Table 2).

**Table 5:** Symbiotic properties of partially effective and ineffective mutants.

Inoculum strain	Genotype	Mean wet foliage weight in mg^a^	% effectiveness based on wet weight	Acetylene reduction^b^
none		19.1±3.0^**^	22.2	0
R7A	Wild-type	86.0±32.2	100	100±35
JS24	*msi158*::*lacZ*	59.2±9.8^*^	68.8	51.5±18.2^*^
JS27	Δ [*msi262-263*]:: Ω*kan*	64.2±22.7	74.6	72.6±33.5
JS42	*nifQ*::*lacZ*	61.9±12^**^	71.9	64.0±15.4^*^
JS28	Δ [*fdxN-fixU*]:: Ω*kan*	17.46±11.6^**^	20.3	0
JS29	Δ [*nifZ-fixU*]:: Ω*kan*	23.6±8.3^**^	27.4	0
JS30	Δ*fixU*:: Ω*kan*	67.5±21.7	78.4	103±22.2

a Mean wet foliage weight of 30 plants ± Standard Deviation. Data were analysed using the Students T-test. ^b^ Percentage of acetylene reduction relative to R7A. Nitrogen fixation activity was measured as the amount of C_2_H_2_ reduced (µmol h^−1^) per plant root for 10 plants ± standard deviation. Data were analysed using the Students T-test. A single asterisk represents *P*<0.05 and two asterisks *P*<0.005 when compared to R7A.

*msi158* encodes an outer membrane porin with strong similarity to members of the *ompW* family (COG3047). While only moderate differences in wet weights were observed between seedlings inoculated with the wild-type and strain JS24 (*msi158*::*lacZ*) (Table 5), plants infected with the mutant were pale yellow-green in appearance at six weeks post-inoculation in comparison to the wild-type. The plasmid pJS105, containing *msi158* and the preceding 392 bp, restored JS24 to a wild-type phenotype. msi036 also encodes a porin (Omp2, COG3452) and is preceded by a NifA-regulated promoter. Msi036 bears no sequence similarity to Msi158. The *msi036*::*lacZ* mutant JS25 was fully effective. To determine if *msi036* and *msi158* were partially functionally redundant, a double mutant JS26 (Δ*msi036*:: Ω*kan msi158*::*lacZ*) was constructed and showed a symbiotic phenotype indistinguishable from that observed for the *msi158* mutant.

The *msi262* (*iscN*; COG0316) and *msi263* (*iscU*; COG0822) genes were deleted by marker exchange, producing the double mutant JS27 (Δ [*msi262-msi263*]:: Ω*kan*). The mutant showed a partial defect in nitrogen fixation (Table 5). The plasmid pJS106, which contained only *msi262* and the preceding 739-bp non-coding region, restored JS27 to the wild-type symbiotic phenotype, indicating that only *msi262* was required for a fully effective symbiosis.

*fdxN, nifZ, fixU*, and *msi351* are located within the *nifB-fdxN-nifZ-fixU-msi351* cluster. To determine whether these genes have a symbiotic role, three marker exchange mutants, designated JS28 (Δ [*fdxN-nifZ-fixU*]:: Ω*kan*), JS29 (Δ [*nifZ-fixU*]:: Ω*kan*) and JS30 (Δ*fixU*:: Ω*kan*), together with JS31 (*msi351*::*lacZ*) were constructed and their symbiotic phenotypes determined. The *msi351*::*lacZ* mutant JS31 was fully effective. JS28 and JS29 displayed an ineffective phenotype, whereas JS30 appeared partially effective. Plants inoculated with JS30 were slightly pale in appearance and displayed a slight reduction in average wet shoot weight; however no difference in acetylene reduction was observed (Table 5). Complementation analysis was performed to determine if both *fdxN* and *nifZ* were required for a fully effective symbiosis using a series of three plasmids. pJS107 contained the *nifB* promoter region and the *nifB-fdxN-nifZ-fixU* cluster with an in-frame deletion within *nifB* and complemented JS28 and JS29 as expected. Plasmid pJS108 contained an in-frame deletion that removed *nifB* and *fdxN*, leaving *nifZ* and *fixU* intact, and complemented JS29 but not JS28. Plasmid pJS109 contained an in-frame deletion that fused the 5′ end of *nifB* to the 3′ end of *nifZ* leaving only *fixU* intact. This plasmid did not complement the JS28 or JS29 mutants but complemented JS30. These results indicated that all three genes were required for a fully effective phenotype, although FixU appeared to exert a very slight influence on nitrogen fixation.

As described above, *prxS* encodes a NifA-regulated peroxiredoxin but is not required for an effective symbiosis. Another NifA-regulated gene present on ICE*Ml*Sym^R7A^, *ccpR* (*msi380*), encodes a cytochrome C peroxidase (COG1858). A *ccpR*::*lacZ* mutant JS32 was Fix^+^ on *L. corniculatus*. In order to determine if functional redundancy existed between *prxS* and *ccpR,* a double mutant JS33 (Δ*prxS ccpR*::*lacZ*) was constructed. The double mutant also formed Fix^+^ nodules.

### Expression and symbiotic phenotypes of genes not preceded by NifA-regulated promoters

In addition to the NifA-regulated operons, the expression of a selection of ICE*Ml*Sym^R7A^ genes encoding metabolic functions and an ABC transporter was examined. The genes were chosen because preliminary screening of *lacZ* expression from IDM mutants showed the genes were expressed at higher levels in 14-day-old nodules than in G/RDM broth cultures (ß-galactosidase activity of less than 20 Miller Units in broth culture for all mutants). This suggested these genes may be subject to symbiosis-specific regulation. The genes selected included *msi083*, which encodes the beta subunit of transketolase, *metE* (*msi160*; 5-methyltetrahydropteroyltriglutamate-homocysteine methyltransferase), *metK* (*msi166*; S-adenosylmethionine synthetase) *pepM* (*msi327*; phosphoenolpyruvate phosphomutase), *msi260* (putative diaminobutyrate-2-oxoglutarate transaminase), *aatA* (*msi326*; aspartate aminotransferase), *asnB* (*msi323*; asparagine synthetase) and *exsA* (*msi339*). *exsA* encodes a MsbA-like saccharide-exporting ABC transporter similar to *S. meliloti* ExsA (71% amino acid identity) that is involved in the export of the exopolysaccharide succinoglycan [27]. The *nifQ* gene (*msi336*) was also selected. *nifQ* is located with the *msi338-msi337-nifQ* gene cluster. Homologs of *msi337* (*fdxB*) and *msi338* are associated with *nif* and *fix* gene clusters preceded by NifA-regulated promoters in some diazotrophs, but a NifA-regulated promoter does not precede the ICE*Ml*Sym^R7A^ cluster. The IDM mutants of *msi083, metE, metK, pepM, msi260, aatA, asnB, exsA* and *nifQ* were designated JS35, JS36 JS37, JS38, JS39, JS42, JS44 and JS45 respectively. *msi337* and *msi338* were inactivated by marker exchange mutagenesis producing strains JS47 and JS48.

With the exception of the *nifQ* (*msi339*), *msi337* and *msi338* mutants which formed partially effective nodules compared to R7A (Table 5), all of the mutants formed a fully effective symbiosis. The *nifQ* gene was amplified by PCR and cloned adjacent to the *nptII* promoter in pFAJ1708 producing plasmid pJS110. This plasmid complemented JS45, JS47 and JS48 to a fully Fix^+^ phenotype, indicating that *nifQ* was the only gene required within the *msi338-msi337-nifQ* cluster for an effective symbiosis.

ß-galactosidase assays performed on extracts from 14-day-old root nodules formed by IDM mutants revealed that, with the exception of *msi083*, all of the genes were poorly expressed in the *nifA2* mutant background, but were strongly expressed in the wild-type and *nifA1* mutant backgrounds. *msi083* was strongly expressed in all three backgrounds (Table 4).

## Discussion

**Figure 3:**
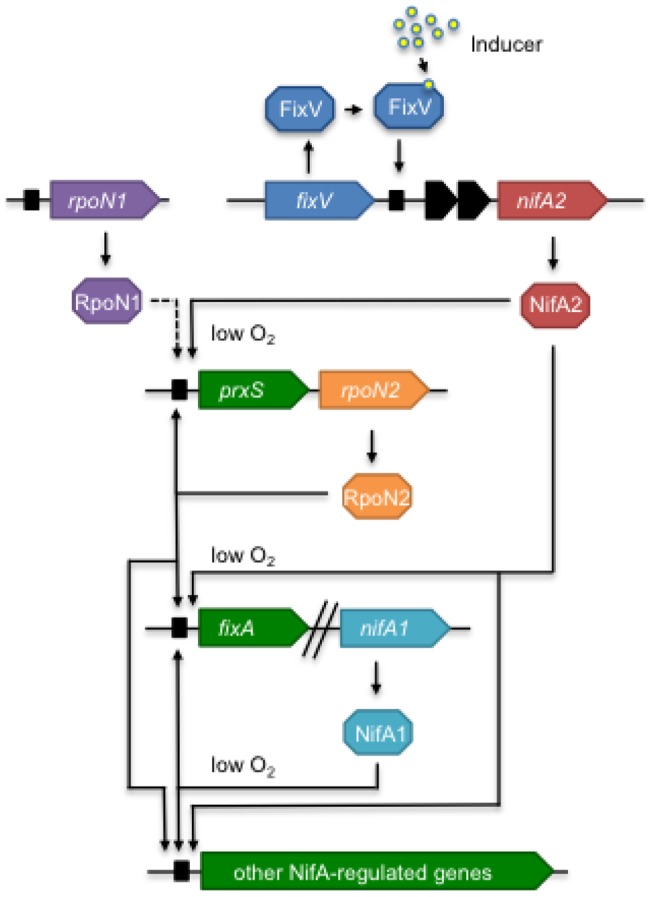
Model for the regulatory network governing symbiotic nitrogen fixation in *M. loti*. In response to an inducer molecule (possibly an inositol derivative), FixV activates expression of *nifA2*. NifA2, in conjunction with RpoN1 or basal levels of RpoN2 produced from an unknown promoter, activates expression of the *prxS-rpoN2* and *fixABCX-nifA1* operons. NifA2 and NifA1, in conjunction with the increased levels of RpoN2, then activate expression of operons required for the production of functional nitrogenase along with other NifA-regulated operons that encode auxiliary metabolic functions.

Our results show that the regulators FixJ, FixK and RegR that initiate symbiotic gene expression in other rhizobia are not required for symbiotic nitrogen fixation in *M. loti*. Instead *M. loti* has evolved a different mechanism for the activation of *nifA* expression. Although *nifA1* encodes a functional NifA protein and is in an identical genomic context to the sole *nifA* gene in several other rhizobial species, it is under the regulation of the product of the second *nifA* gene, *nifA2*. The *nifA2* gene in turn is under the regulation of a novel regulator FixV that is a member of the LacI/GalR family. A model for the regulatory network governing symbiotic nitrogen fixation in *M. loti* is shown in Fig. 3.

The *fixV* gene is located upstream of *nifA2* and the 558-bp intergenic region was found to contain gene fragments homologous to an ICE*Ml*Sym^R7A^ gene *msi119* that encodes a sugar epimerase. Homology was detectable at both the nucleotide and amino acid levels. Analysis of a series of mutants with a nested set of promoter deletions showed that sequences required for *nifA2* expression were located downstream of *fixV* but upstream of the gene fragments. Bioinformatic analysis revealed that homologs of *msi119* were located downstream of *fixV* homologs in *M. ciceri, R. etli, R. leguminosarum* and *Mesorhizobium* sp. strain CJ1 (Fig. 2). Taken together, these results suggest that expression of *nifA2* was placed under FixV control by a translocation event involving *fixV* and a downstream promoter that it regulates. Furthermore it seems likely that FixV responds to a carbohydrate signal to initiate *nifA2* transcription. The LacI/GalR family of transcriptional regulators consist of an N-terminal helix-turn-helix DNA-binding domain and a C-terminal ligand-binding domain that is structurally homologous to periplasmic sugar-binding proteins [28], [29], [30]. While most family members are repressors, a few members are activators. The most closely related ortholog outside of the analogous copy on the *M. loti* MAFF303099 symbiosis island was found on the symbiosis island of *M. ciceri* bv. *biserrulae* WSM1271 (76% amino-acid identity) where *fixV* is directly upstream of a *msi119* homolog and close to *nifA2*. The *msi119* homolog is also directly downstream of and divergently transcribed from *nodD2* as it is in R7A and MAFF303099 (Fig. 2). It appears possible that WSM1271 represents the ancestral genetic organisation and that a series of recombination events may have led to the arrangement observed in R7A and MAFF303099. The next strongest homologs of FixV (approx. 60% identity, 75% similarity over entire protein) are encoded on the *M. loti* MAFF303099 chromosome, and on plasmids in *S. meliloti, Rhizobium* sp. NGR234, *R. etli* and *R. leguminosarum*. They are located adjacent to a *mocDEF* cluster involved in rhizopine catabolism. In the case of *R. etli* and *R. leguminosarum*, the *msi119* homolog is downstream of the *fixV* homolog. Rhizopine is L-3-O-methyl-*scyllo*-inosamine, a derivative of inositol [31], and so these observations strongly suggest that Msi119 homologs are involved in catabolism of an inositol derivative. Furthermore, Msi119 is a member of pfam01261, defined by the presence of a TIM alpha/beta barrel structure that is found in xylose isomerase, endonuclease IV and in the N termini of bacterial *myo*-inositol catabolism proteins. Inositol derivatives play a wide variety of roles in plants and *myo-*inositol is one of the more abundant non-structural carbohydrates in soybean nodules, where it is largely localized to the peribacteroid space [32]. It thus seems possible that the metabolite FixV senses to activate expression of *nifA2* is a derivative of inositol.

A combination of mutagenesis and complementation analysis of the *prxS-rpoN2* operon showed that *rpoN2* but not *prxS* was essential for an effective symbiosis. In contrast, *rpoN1* was required for C4-dicarboxylate usage in free-living *M. loti* but was not required for symbiosis. These results are similar to those obtained with *R. etli*, except that nodules formed by the *R. etli rpoN2* mutant show a low level of nitrogen fixation activity [19]. The *prxS-rpoN2* operon is preceded by an RpoN-dependent promoter and a NifA UAS sequence. It is interesting to note that significant expression of the *prxS-rpoN2* operon was observed in bacteroids formed by JS06A (*prxS*::*lacZ*) and JS05B (*rpoN2*::*lacZ*), given that both these mutants were ineffective as a result of inactivation of *rpoN2*. This activity was approximately 17% (*rpoN2*::*lacZ*) or 25% (*prxS*::*lacZ* ) of that obtained with a *prxS^+^::lacZ cis*-merodiploid strain (JS07) where the *prxS-rpoN2* operon remains intact. For *R. etli,* expression studies have suggested that a weak symbiosis-specific promoter is located between the end of *prxS* and start of *rpoN2*. Symbiosis-specific expression from this promoter initiates *rpoN-*independent expression of the *prxS-rpoN2* operon in an *rpoN1* mutant background [21]. However in nodules formed by *M. loti* R7A, the expression of both *prxS* and *rpoN2* in the *nifA2* mutant background appeared wholly dependent on NifA2 (Table 4). Nevertheless, the fact that RpoN1 is not required for symbiotic nitrogen fixation indicates that there must be some NifA-independent expression of *rpoN2* in *M. loti*. Whether this is basal expression from the promoter upstream of *prxS* or expression from a weak promoter between *prxS* and *rpoN2*, as is the case in *R. etli*, remains to be determined.

It is striking that in *R. etli, R. leguminosarum* and *M. loti*, the strongly expressed and symbiotically essential *nifH* promoter deviates from the −24/−12 consensus at the critical −12 region, with an A instead of the highly conserved C. An A at −12 was found in only 9 out of 186 potential RpoN-regulated promoters identified *in silico* from 44 species belonging to the *Rhizobiales* [33]. The RpoN-dependent promoter of the *fdxN* gene of *B. japonicum* also has an A at the −12 position and this promoter is active in *B. japonicum* but not *E. coli*, unlike other *B. japonicum* NifA-regulated promoters [34]. The RpoN1 and RpoN2 proteins of *R. sphaeroides* show specificity to transcribe a particular set of genes that is due in part to the particular nucleotide at the −11 position of the promoter and in part because they only function with their cognate activators [35]. The expression of the *prxS-rpoN2* promoter observed in *rpoN2* (and *rpoN2/nifA1*) mutant background(s) rules out the possibility that only RpoN2 and not RpoN1 can interact with NifA2. Hence it seems possible that in *M. loti* only RpoN2 and not RpoN1 recognizes the atypical *nifH* promoter.

The construction of IDM mutants allowed us to determine both expression of the mutated gene in its normal genomic context and the symbiotic phenotype of the mutant. The expression patterns of the putative NifA-regulated operons in the *nifA2* and *nifA1* mutant backgrounds were consistent with their direct activation by NifA-RpoN. The results also confirmed that *nifA1* was not required for expression of any of the NifA*-*regulated genes located on ICE*Ml*Sym^R7A^. Several ICE*Ml*Sym^R7A^-encoded genes not associated with NifA-regulated promoters (*msi083, metE, metK, pepM, msi260, aatA, asnB, nifQ, exsA*) were also strongly expressed in nodules but, with the exception of *msi083*, were not expressed in the *nifA2* mutant background. It is highly unlikely that their expression is directly activated by NifA. The fact that *msi083* and *nifA2* expression was readily detected in the nodules formed by *nifA2* mutants indicates that expression of these genes would have been detected had it occurred. It seems likely that other factors influenced by NifA expression under symbiotic conditions such as nitrogen and carbon fluxes and oxygen tension induce the expression of these genes in functional nodules. These results contrast with those observed when microarray and proteome analysis was performed on RNA and protein extracted at 11 days post-inoculation from *Phaseolus vulgaris* nodules formed by wild-type *R. etli* strain CFN42 and a *nifA* mutant. This analysis revealed only five genes that were not preceded by RpoN and/or NifA regulatory elements that were down-regulated in the *nifA* mutant versus the wild-type under symbiotic conditions [36]. None of these genes corresponded to those found to be down-regulated in *Lotus* nodules in the current study.

In common with studies of other rhizobia, we observed that many genes that were strongly expressed in nodules did not produce an overt symbiotic phenotype when mutated. However, of the genes not previously shown to be required for symbiosis, *msi158* and *nifZ* that are regulated by NifA and *nifQ* that lacks a NifA-regulated promoter were found to be required for a fully effective symbiosis. The *msi158* mutant formed partially effective nodules and the plants were yellowish, indicating nitrogen deficiency. The *msi158* gene encodes an outer membrane protein of the OmpW family (COG3047) that shares strong similarity with the gene products of *y4MB* present on pNGR234a of *Rhizobium* sp. NGR234 and *bll1766* from the symbiotic region of *B. japonicum.* NifA-regulated promoters are also located upstream of these two orthologs [5], [11], [37]. A *bll1766* mutant formed normal nitrogen-fixing nodules on soybean [37]. However a strongly conserved *bll1766* ortholog (*blr1311*) is located elsewhere on the *B. japonicum* USDA110 genome [38]. No orthologs are present in the *S. meliloti* 1021, *R. leguminosarum* bv. *viciae* strain 3841, *R. leguminosarum* bv. *trifolii* WSM1325, or *R. etli* CFN42 genomes, or in *M. loti* MAFF303099 outside of the symbiosis island [5], [39], [40], [41], [42], [43], [44]. The *E. coli* OmpW protein forms an eight-stranded ß-barrel with a hydrophobic channel and may be involved in the transport of small hydrophobic molecules across the bacterial outer membrane [45]. In *Salmonella enterica* serovar Typhimurium, the *ompW* gene is part of the SoxRS regulon that protects against oxidative stress and it has been suggested that the porin functions as an efflux channel for toxic compounds generated during oxidative stress [46]. A similar role in *M. loti* would make *msi158* the third member of the NifA-RpoN regulon together with *prxS* and *ccpR* likely involved in protection against reactive oxygen species.

The different rhizobial species vary considerably in the complement of *nif* genes that they share with the paradigm nitrogen-fixing microorganism, the free-living diazotroph *Klebsiella pneumoniae*, and it is apparent that the nitrogenase assembly machinery is to an extent species-specific in rhizobia (reviewed in [47]). For example, the *nifB-fdxN-nifZ-fixU-msi351* cluster found in *M. loti* is present to varying extents in other rhizobia: it is complete in *R. etli* CFN42 and *M. ciceri* bv *biserrulae* WSM1271, missing *msi351* in *Rhizobium* sp. strain NGR234, missing *nifZ* and *msi351* in *S. meliloti* 1021 and *Bradyrhizobium* species ORS278 and BTAi1 (although *nifZ* is located elsewhere in the latter two species), while in *R. leguminosarum* only *nifB* is present [5], [38], [39], [40], [41], [42], [43], [44], [48], [49]. We showed that mutations within the ICE*Ml*Sym^R7A^ *nifZ* and *fdxN* genes abolished nitrogen fixation. The *nifZ* gene is found in several diazotrophs and is involved in maturation of the Mo-Fe protein [50]. The FdxN protein is thought to serve in the pathway of electron transfer to nitrogenase. In *S. meliloti* mutations within *fdxN* also completely abolish nitrogen fixation [51]. The function of *fixU* (also called *nifT* in some diazotrophs) is unknown and inactivation of *nifT* in *K. pneumoniae* has no obvious effects on nitrogen fixation [52], [53]. Our results show that active FixU is required for optimal N fixation in *M. loti* under the growth conditions used.

The *msi338-msi337-nifQ* gene cluster is not preceded by a NifA-regulated promoter although a NifA-regulated promoter is present upstream of a *nifQ* fragment that precedes *msi332* on ICE*Ml*Sym^R7A^. Homologs of *msi337* (*fdxB*) and *msi338* are located within or adjacent to *nif* and *fix* clusters preceded by NifA-regulated promoters in *S. meliloti, R. etli* and *B. japonicum. R. leguminosarum* possesses *fdxB* but not *msi338* while *nifQ* is absent from *S. meliloti* and *R. leguminosarum* [38], [39], [40], [41], [42], [43], [44]. Homologs of Msi338 are also encoded within nitrogen fixation gene clusters of a wide range of bacteria [54]. NifQ participates as a molybdenum donor for FeMoCo biosynthesis [55]. Our results showed that *nifQ* was required for a fully effective symbiosis, in contrast to the situation in *Rhizobium* sp. strain NGR234 where mutation of *nifQ* had no effect on symbiotic nitrogen fixation [56]. The lack of a symbiotic defect in *msi337* and *msi338* mutants may reflect functional redundancy as probable orthologs of these genes are present in the *msi276-msi275-msi274* gene cluster that is preceded by a NifA-regulated promoter. Consistent with this, a mutant strain JS50 (Δ [*msi274-msi276*]:: Ω*kan*) in which all three genes were deleted formed Fix^+^ nodules.

The *msi262* and *msi263* genes were renamed *iscN* and *iscU* respectively and are likely involved in the production of iron-sulfur clusters for nitrogenase. Msi262 shows 71% identity to the *R. etli iscN* gene product that is thought to act as a scaffold protein for Fe-S biosynthesis. Mutants of *R. etli* defective in *iscN* showed a 90% reduction in nitrogen fixation [57]. Msi263 is a member of the IscU protein family (COG0822). These proteins are similar to the N-terminal region of NifU and are also thought to play a scaffolding role in Fe-S cluster formation. As suggested for *R. etli*, it seems likely that the IscN and IscU homologs are partially functionally redundant. However the *iscN-iscU* double mutant was partially effective, suggesting that *M. loti* may harbor additional genes that can at least partially complement their function.

In summary, a novel regulator FixV together with NifA2 were identified as key regulators of genes required for nodule function in *M. loti*, with FixV activating *nifA2* expression possibly in response to a plant-produced inositol derivative (Fig. 3). Many genes encoded on ICE*Ml*Sym^R7A^ were strongly expressed in nodules in a NifA2-dependent manner but not free-living rhizobia. Nevertheless most of these genes were not required for symbiotic nitrogen fixation. It seems likely that some of these genes have functional homologues elsewhere in the genome and that bacteroid metabolism may be sufficiently plastic to adapt to loss of various enzymatic functions.

## Materials and Methods

### Bacterial strains, plasmids and growth conditions

The wild-type *M. loti* strain used in this study was R7A, a field reisolate of ICMP 3153 (NZP2238) [2]. Mutant strains constructed in the R7A, JS01 (R7AΔ*nifA1*) and JS02 (R7AΔ*nifA2*) backgrounds are described in Table 2. Plasmids are listed in Table 3. *M. loti* strains were grown at 28°C in TY [58] or in rhizobium defined medium with 10 mM glucose (G/RDM) or 10 mM succinate (S/RDM) as previously described [59]. *Escherichia coli* strain S17-1 [60] was used for cloning and as the donor for biparental matings. It was cultured in LB or TY medium. Antibiotics were used at the following concentrations: for *E. coli*, tetracycline 15 µg mL^−1^, kanamycin 50 µg mL^−1^ and gentamicin 25 µg mL^−1^; and for *M. loti* tetracycline 2 µg mL^−1^, neomycin 200 µg mL^−1^, and gentamicin 50 µg mL^−1^.

### DNA manipulations

Plasmid DNA preparations DNA cloning, transformation of DNA into *E. coli* and Southern hybridisations were carried out using established techniques [61]. Genomic DNA was extracted as described previously [2]. PCR was performed using an Expand HiFi PCR kit (Roche).

### Construction of mutants and *lacZ* promoter fusions

Insertion duplication mutants (IDM) and *cis*-merodiploid (CMD) *lacZ* fusions were constructed using the suicide vector pFUS2 [62]. Oligonucleotide primer pairs incorporating restriction sites were used to amplify 350–500 bp regions which comprised either intragenic regions of the target genes to create IDM mutants or the promoter region and 5′ end to create strains containing promoter-*lacZ* fusions, leaving the associated gene and its promoter region intact. PCR products were then cloned into pFUS2 adjacent to its promoterless *lacZ* gene and confirmed by sequencing using a *lacZ*-specific primer. pFUS2 constructs were transferred into *M. loti* by conjugation from *E. coli* strain S17-1 donors as described [7] and transconjugants were passaged three times on selective media and then confirmed by Southern hybridization.

Marker exchange mutants were constructed by replacing the gene of interest with the ΩKan interposon [63]. Oligonucleotide primer pairs were designed to amplify 1-kb regions that flanked the target gene and they contained restriction enzyme sites to facilitate cloning. The PCR products were digested with appropriate enzymes and ligated into pIJ3200 along with the ΩKan interposon from pHP45ΩKan [63]. The resulting plasmid was confirmed by DNA sequencing and transferred into R7A by conjugation. Recombination was then forced by plasmid incompatibility using pPH1JI [64] and the mutant confirmed by Southern hybridization. pPH1JI was then removed from the strain by introducing pLAFR1 and an isolate that had lost pLAFR1 was selected as described previously [7].

Markerless deletion mutants of *M. loti* were constructed using the suicide vector pJQ200SK [65]. One-kilobase regions that flanked the gene were amplified by PCR using primers that included restriction endonuclease sites for cloning. The PCR products were digested and ligated into pJQ200SK. Clones were confirmed by DNA sequencing and then transferred to R7A by conjugation, followed by selection for gentamicin-resistant clones. Integration at the correct site was confirmed by Southern hybridization. Loss of sucrose sensitivity, caused by loss of the *sacB* gene located on pJQ200SK, was used to select clones that had undergone a second recombination event that removed the vector. Southern hybridization was used to confirm the final deletion derivatives.

### Plant assays

Plant studies were performed using *L. corniculatus* cv. Goldie as previously described [66]. Surface-sterilized seeds were germinated on 0.8% water agar. Seedlings were planted on Jensen's agar slopes in glass test-tubes. For testing mutants for symbiotic effectiveness, 15 plants were inoculated by addition of 100 µl of a cell suspension containing approximately 10^6^ cells. Seedlings were cultivated under environmental conditions of 70% humidity, 25°C, 16 h light, 14°C, 8 h dark. Plants were harvested at six weeks post-inoculation and the effectiveness of the symbiosis determined by visual inspection and by measuring the wet weight of foliage above the first cotyledonary node [66]. Nitrogenase assays were performed on nodulated roots harvested from 15 seedlings as described previously [67]. β-galactosidase assays were performed on bacteroid suspensions as previously described [68] using bacteroid suspensions prepared from nodules harvested 14 days post-inoculation from six plants per inoculum.

## References

[1] Sullivan JT, Ronson CW (1998) Evolution of rhizobia by acquisition of a 500-kb symbiosis island that integrates into a phe-tRNA gene. Proc Natl Acad Sci USA 95: 5145–5149. 9560243 10.1073/pnas.95.9.5145 PMC20228

[2] Sullivan JT, Patrick HN, Lowther WL, Scott DB, Ronson CW (1995) Nodulating strains of *Rhizobium loti* arise through chromosomal symbiotic gene transfer in the environment. Proc Natl Acad Sci USA 92: 8985–8989. 7568057 10.1073/pnas.92.19.8985 PMC41092

[3] Ramsay JP, Sullivan JT, Stuart GS, Lamont IL, Ronson CW (2006) Excision and transfer of the *Mesorhizobium loti* R7A symbiosis island requires an integrase IntS, a novel recombination directionality factor RdfS, and a putative relaxase RlxS. Mol Microbiol 62: 723–734. 17076666 10.1111/j.1365-2958.2006.05396.x

[4] Wozniak RA, Waldor MK (2010) Integrative and conjugative elements: mosaic mobile genetic elements enabling dynamic lateral gene flow. Nat Rev Microbiol 8: 552–563. 20601965 10.1038/nrmicro2382

[5] Kaneko T, Nakamura Y, Sato S, Asamizu E, Kato T, et al (2000) Complete genome structure of the nitrogen-fixing symbiotic bacterium *Mesorhizobium loti* . DNA Res 7: 331–338. 11214968 10.1093/dnares/7.6.331

[6] Sullivan JT, Trzebiatowski JR, Cruickshank RW, Gouzy J, Brown SD, et al (2002) Comparative sequence analysis of the symbiosis island of *Mesorhizobium loti* strain R7A. J Bacteriol 184: 3086–3095. 12003951 10.1128/JB.184.11.3086-3095.2002 PMC135072

[7] Hubber A, Vergunst AC, Sullivan JT, Hooykaas PJJ, Ronson CW (2004) Symbiotic phenotypes and translocated effector proteins of the *Mesorhizobium loti* strain R7A VirB/D4 type IV secretion system. Mol Microbiol 54: 561–574. 15469524 10.1111/j.1365-2958.2004.04292.x

[8] Fischer HM (1994) Genetic regulation of nitrogen fixation in rhizobia. Microbiol Rev 58: 352–386. 7968919 10.1128/mr.58.3.352-386.1994 PMC372973

[9] Batut J, Terzaghi B, Gherardi M, Huguet M, Terzaghi E, et al (1985) Localization of a symbiotic *fix* region on *Rhizobium meliloti* pSym megaplasmid more than 200 kilobases from the *nod-nif* region. Mol Gen Genet 199: 232–239.

[10] David M, Domergue O, Pognonec P, Kahn D (1987) Transcription patterns of *Rhizobium meliloti* symbiotic plasmid pSym: identification of *nifA*-independent *fix* genes. J Bacteriol 169: 2239–2244. 2437100 10.1128/jb.169.5.2239-2244.1987 PMC212141

[11] Freiberg C, Fellay R, Bairoch A, Broughton WJ, Rosenthal A, et al (1997) Molecular basis of symbiosis between *Rhizobium* and legumes. Nature 387: 394–401. 9163424 10.1038/387394a0

[12] González V, Bustos P, Ramírez-Romero MA, Medrano-Soto A, Salgado H, et al (2003) The mosaic structure of the symbiotic plasmid of *Rhizobium etli* CFN42 and its relation to other symbiotic genome compartments. Genome Biol 4: R36. 12801410 10.1186/gb-2003-4-6-r36 PMC193615

[13] Scott KF, Rolfe BG, Shine J (1983) Biological nitrogen fixation: primary structure of the *Rhizobium trifolii* iron protein gene. DNA 2: 149–155. 6307623 10.1089/dna.1983.2.149

[14] Nukui N, Minamisawa K, Ayabe S, Aoki T (2006) Expression of the 1-aminocyclopropane-1-carboxylic acid deaminase gene requires symbiotic nitrogen-fixing regulator gene *nifA2* in *Mesorhizobium loti* MAFF303099. Appl Environ Microbiol 72: 4964–4969. 16820494 10.1128/AEM.02745-05 PMC1489367

[15] Sullivan JT, Brown SD, Yocum RR, Ronson CW (2001) The *bio* operon on the acquired symbiosis island of *Mesorhizobium* sp. strain R7A includes a novel gene involved in pimeloyl-CoA synthesis. Microbiology 147: 1315–1322. 11320134 10.1099/00221287-147-5-1315

[16] Bauer E, Kaspar T, Fischer HM, Hennecke H (1998) Expression of the *fixR-nifA* operon in *Bradyrhizobium japonicum* depends on a new response regulator, RegR. J Bacteriol 180: 3853–3863. 9683482 10.1128/jb.180.15.3853-3863.1998 PMC107369

[17] Martínez M, Palacios JM, Imperial J, Ruiz-Argüeso T (2004) Symbiotic autoregulation of *nifA* expression in *Rhizobium leguminosarum* bv. *viciae* . J Bacteriol 186: 6586–6594. 15375140 10.1128/JB.186.19.6586-6594.2004 PMC516587

[18] Benhassine T, Fauvart M, Vanderleyden J, Michiels J (2007) Interaction of an IHF-like protein with the *Rhizobium etli nifA* promoter. FEMS Microbiol Lett 271: 20–26. 17403047 10.1111/j.1574-6968.2007.00699.x

[19] Michiels J, Van Soom T, D'Hooghe I, Dombrecht B, Benhassine T, et al (1998) The *Rhizobium etli rpoN* locus: DNA sequence analysis and phenotypical characterization of *rpoN, ptsN*, and *ptsA* mutants. J Bacteriol 180: 1729–1740. 9537369 10.1128/jb.180.7.1729-1740.1998 PMC107084

[20] Michiels J, Moris M, Dombrecht B, Verreth C, Vanderleyden J (1998) Differential regulation of *Rhizobium etli rpoN2* gene expression during symbiosis and free-living growth. J Bacteriol 180: 3620–3628. 9658006 10.1128/jb.180.14.3620-3628.1998 PMC107331

[21] Dombrecht B, Heusdens C, Beullens S, Verreth C, Mulkers E, et al (2005) Defence of *Rhizobium etli* bacteroids against oxidative stress involves a complexly regulated atypical 2-Cys peroxiredoxin. Mol Microbiol 55: 1207–1221. 15686565 10.1111/j.1365-2958.2005.04457.x

[22] Kullik I, Fritsche S, Knobel H, Sanjuan J, Hennecke H, et al (1991) *Bradyrhizobium japonicum* has two differentially regulated, functional homologs of the sigma-54 gene (RpoN). J Bacteriol 173: 1125–1138. 1991712 10.1128/jb.173.3.1125-1138.1991 PMC207233

[23] Ronson CW, Nixon BT, Albright LM, Ausubel FM (1987) *Rhizobium meliloti ntrA* (*rpoN*) gene is required for diverse metabolic functions. J Bacteriol 169: 2424–2431. 3034856 10.1128/jb.169.6.2424-2431.1987 PMC212082

[24] Clark SRD, Oresnik IJ, Hynes MF (2001) RpoN of *Rhizobium leguminosarum* bv. *viciae* strain VF39SM plays a central role in FnrN-dependent microaerobic regulation of genes involved in nitrogen fixation. Mol Gen Genet 264: 623–633. 11212917 10.1007/s004380000348

[25] Uchiumi T, Ohwada T, Itakura M, Mitsui H, Nukui N, et al (2004) Expression islands clustered on the symbiosis island of the *Mesorhizobium loti* genome. J Bacteriol 186: 2439–2448. 15060047 10.1128/JB.186.8.2439-2448.2004 PMC412173

[26] Altschul SF, Gish W, Miller W, Myers EW, Lipman DJ (1990) Basic local alignment search tool. J Mol Biol 215: 403–410. 2231712 10.1016/S0022-2836(05)80360-2

[27] Becker A, Küster H, Niehaus K, Pühler A (1995) Extension of the *Rhizobium meliloti* succinoglycan biosynthesis gene cluster: identification of the *exsA* gene encoding an ABC transporter protein, and the *exsB* gene which probably codes for a regulator of succinoglycan biosynthesis. Mol Gen Genet 249: 487–497. 8544814 10.1007/BF00290574

[28] Fukami-Kobayashi K, Tateno Y, Nishikawa K (2003) Parallel evolution of ligand specificity between LacI/GalR family repressors and periplasmic sugar-binding proteins. Mol Biol Evol 20: 267–277. 12598694 10.1093/molbev/msg038

[29] Nguyen CC, Saier MH (1995) Phylogenetic, structural and functional analyses of the LacI-GalR family of bacterial transcription factors. FEBS Lett 377: 98–102. 8543068 10.1016/0014-5793(95)01344-x

[30] Weickert MJ, Adhya S (1992) A family of bacterial regulators homologous to Gal and Lac repressors J Biol Chem. 267: 15869–15874. 1639817

[31] Murphy PJ, Heycke N, Trenz SP, Ratet P, de Bruijn FJ, et al (1988) Synthesis of an opine-like compound, a rhizopine, in alfalfa nodules is symbiotically regulated. Proc Natl Acad Sci USA 85: 9133–9137. 2848255 10.1073/pnas.85.23.9133 PMC282678

[32] Tejima K, Arima Y, Yokoyama T, Sekimoto H (2003) Composition of amino acids, organic acids, and sugars in the peribacteroid space of soybean root nodules. Soil Sci Plant Nutr 49: 293–247.

[33] Dombrecht B, Marchal K, Vanderleyden J, Michiels J (2002) Prediction and overview of the RpoN-regulon in closely related species of the Rhizobiales. Genome Biol 3: 0076.0071. 10.1186/gb-2002-3-12-research0076 PMC151178 12537565

[34] Hauser F, Pessi G, Friberg M, Weber C, Rusca N, et al (2007) Dissection of the *Bradyrhizobium japonicum* NifA+sigma54 regulon, and identification of a ferredoxin gene (*fdxN*) for symbiotic nitrogen fixation. Mol Genet Genomics 278: 255–271. 17569992 10.1007/s00438-007-0246-9

[35] Poggio S, Osorio A, Dreyfus G, Camarena L (2006) Transcriptional specificity of RpoN1 and RpoN2 involves differential recognition of the promoter sequences and specific interaction with the cognate activator proteins. J Biol Chem 281: 27205–27215. 16854992 10.1074/jbc.M601735200

[36] Salazar E, Díaz-Mejía JJ, Moreno-Hagelsieb G, Martínez-Batallar G, Mora Y, et al (2010) Characterization of the NifA-RpoN regulon in *Rhizobium etli* in free life and in symbiosis with *Phaseolus vulgaris* . Appl Environ Microbiol 76: 4510–4520. 20453139 10.1128/AEM.02007-09 PMC2897426

[37] Caldelari Baumberger I, Fraefel N, Göttfert M, Hennecke H (2003) New NodW- or NifA-regulated *Bradyrhizobium japonicum* genes. Mol Plant-Microbe Interact 16: 342–351. 12744463 10.1094/MPMI.2003.16.4.342

[38] Kaneko T, Nakamura Y, Sato S, Minamisawa K, Uchiumi T, et al (2002) Complete genomic sequence of nitrogen-fixing symbiotic bacterium *Bradyrhizobium japonicum* USDA110. DNA Res 9: 189–197. 12597275 10.1093/dnares/9.6.189

[39] Finan TM, Weidner S, Wong K, Buhrmester J, Chain P, et al (2001) The complete sequence of the 1,683-kb pSymB megaplasmid of the N_2_-fixing endosymbiont *Sinorhizobium meliloti* . Proc Natl Acad Sci USA 98: 9889–9894. 11481431 10.1073/pnas.161294698 PMC55548

[40] Barnett MJ, Fisher RF, Jones T, Komp C, Abola AP, et al (2001) Nucleotide sequence and predicted functions of the entire *Sinorhizobium meliloti* pSymA megaplasmid. Proc Natl Acad Sci USA 98: 9883–9888. 11481432 10.1073/pnas.161294798 PMC55547

[41] Capela D, Barloy-Hubler F, Gouzy J, Bothe G, Ampe F, et al (2001) Analysis of the chromosome sequence of the legume symbiont *Sinorhizobium meliloti* strain 1021. Proc Natl Acad Sci USA 98: 9877–9882. 11481430 10.1073/pnas.161294398 PMC55546

[42] Young JP, Crossman LC, Johnston AW, Thomson NR, Ghazoui ZF, et al (2006) The genome of *Rhizobium leguminosarum* has recognizable core and accessory components. Genome Biol 7: R34. 16640791 10.1186/gb-2006-7-4-r34 PMC1557990

[43] González V, Santamaría RI, Bustos P, Hernández-González I, Medrano-Soto A, et al (2006) The partitioned *Rhizobium etli* genome: genetic and metabolic redundancy in seven interacting replicons. Proc Natl Acad Sci USA 103: 3834–3839. 16505379 10.1073/pnas.0508502103 PMC1383491

[44] Reeve W, O'Hara G, Chain P, Ardley J, Bräu L, et al (2010) Complete genome sequence of *Rhizobium leguminosarum* bv. *trifolii* strain WSM1325, an effective microsymbiont of annual Mediterranean clovers. Stand Genomic Sci 2: 347–356. 21304718 10.4056/sigs.852027 PMC3035295

[45] Hong H, Patel DR, Tamm LK, van den Berg B (2006) The outer membrane protein OmpW forms an eight-stranded beta-barrel with a hydrophobic channel. J Biol Chem 281: 7568–7577. 16414958 10.1074/jbc.M512365200

[46] Gil F, Hernández-Lucas I, Polanco R, Pacheco N, Collao B, et al (2009) SoxS regulates the expression of the *Salmonella enterica* serovar Typhimurium *ompW* gene. Microbiology 155: 2490–2497. 19460824 10.1099/mic.0.027433-0

[47] Masson-Boivin C, Giraud E, Perret X, Batut J (2009) Establishing nitrogen-fixing symbiosis with legumes: how many rhizobium recipes? Trends Microbiol 17: 458–466. 19766492 10.1016/j.tim.2009.07.004

[48] Giraud E, Moulin L, Vallenet D, Barbe V, Cytryn E, et al (2007) Legumes symbioses: absence of *nod* genes in photosynthetic bradyrhizobia. Science 316: 1307–1312. 17540897 10.1126/science.1139548

[49] Lucas S, Copeland A, Lapidus A, Cheng JF, Goodwin L, et al. (2011) Complete sequence of chromosome of *Mesorhizobium ciceri* bv. *biserrulae* WSM1271. unpublished Genbank accessions CP002447.1, CP002448.1). Available: http://www.ncbi.nlm.nih.gov/bioproject/48991.

[50] Cotton MS, Rupnik K, Broach RB, Hu Y, Fay AW, et al (2009) VTVH-MCD study of the Δ*nifB*Δ*nifZ* MoFe protein from *Azotobacter vinelandii* . J Am Chem Soc 131: 4558–4559. 19334767 10.1021/ja807525m

[51] Klipp W, Reiländer H, Schlüter A, Krey R, Pühler A (1989) The *Rhizobium meliloti fdxN* gene encoding a ferredoxin-like protein is necessary for nitrogen fixation and is cotranscribed with *nifA* and *nifB* . Mol Gen Genet 216: 293–302. 2747618 10.1007/BF00334368

[52] Harris GS, White TC, Flory JE, Orme-Johnson WH (1990) Genes required for formation of the apoMoFe protein of *Klebsiella pneumoniae* nitrogenase in *Escherichia coli* . J Biol Chem 265: 15909–15919. 2203791

[53] Simon HM, Homer MJ, Roberts GP (1996) Perturbation of *nifT* expression in *Klebsiella pneumoniae* has limited effect on nitrogen fixation. J Bacteriol 178: 2975–2977. 8631690 10.1128/jb.178.10.2975-2977.1996 PMC178037

[54] Buchko GW, Robinson H, Addlagatta A (2009) Structural characterization of the protein cce_0567 from Cyanothece 51142, a metalloprotein associated with nitrogen fixation in the DUF683 family. Biochim Biophys Acta 1794: 627–633. 19336042 10.1016/j.bbapap.2009.01.002 PMC3707797

[55] Hernandez JA, Curatti L, Aznar CP, Perova Z, Britt RD, et al (2008) Metal trafficking for nitrogen fixation: NifQ donates molybdenum to NifEN/NifH for the biosynthesis of the nitrogenase FeMo-cofactor. Proc Natl Acad Sci USA 105: 11679–11684. 18697927 10.1073/pnas.0803576105 PMC2575292

[56] Fumeaux C, Bakkou N, Kopcinska J, Golinowski W, Westenberg DJ, et al (2011) Functional analysis of the *nifQdctA1y4vGHIJ* operon of *Sinorhizobium fredii* strain NGR234 using a transposon with a NifA-dependent read-out promoter. Microbiology 157: 2745–2758. 21719545 10.1099/mic.0.049999-0

[57] Dombrecht B, Tesfay MZ, Verreth C, Heusdens C, Napoles MC, et al (2002) The *Rhizobium etli* gene *iscN* is highly expressed in bacteroids and required for nitrogen fixation. Mol Genet Genomics 267: 820–828. 12207230 10.1007/s00438-002-0715-0

[58] Beringer JE (1974) R factor transfer in *Rhizobium leguminosarum* . J Gen Microbiol 84: 188–198. 4612098 10.1099/00221287-84-1-188

[59] Ronson CW, Astwood PM, Nixon BT, Ausubel FM (1987) Deduced products of C_4_-dicarboxylate transport regulatory genes of *Rhizobium leguminosarum* are homologous to nitrogen regulatory gene products. Nucleic Acids Res 15: 7921–7934. 3671068 10.1093/nar/15.19.7921 PMC306317

[60] Simon R, Priefer U, Pühler A (1983) A broad-host mobilization system for *in vivo* genetic engineering: transposon mutagenesis in gram-negative bacteria. Biotechnology (NY) 1: 784–791.

[61] Sambrook J, Fritsch EF, Maniatis T (1989) Molecular cloning: A laboratory manual. New York, USA: Cold Spring Harbor Laboratory Press.

[62] Antoine R, Alonso S, Raze D, Coutte L, Lesjean S, et al (2000) New virulence-activated and virulence-repressed genes identified by systematic gene inactivation and generation of transcriptional fusions in *Bordetella pertussis* . J Bacteriol 182: 5902–5905. 11004193 10.1128/jb.182.20.5902-5905.2000 PMC94716

[63] Fellay R, Krisch HM, Prentki P, Frey J (1989) Ω-Km – a transposable element designed for *in vivo* insertional mutagenesis and cloning of genes in gram-negative bacteria. Gene 76: 215–226. 2546859 10.1016/0378-1119(89)90162-5

[64] Ruvkun GB, Ausubel FM (1981) A general method for site-directed mutagenesis in prokaryotes. Nature 289: 85–88. 6256652 10.1038/289085a0

[65] Quandt J, Hynes MF (1993) Versatile suicide vectors which allow direct selection for gene replacement in gram-negative bacteria. Gene 127: 15–21. 8486283 10.1016/0378-1119(93)90611-6

[66] Vincent JM (1970) A manual for the practical study of root nodule bacteria. Oxford: Blackwell Scientific Publications.

[67] Hussain AKMA, Jiang Q, Broughton WJ, Gresshoff PM (1999) *Lotus japonicus* nodulates and fixes nitrogen with the broad host range *Rhizobium* sp. NGR234. Plant Cell Physiol 40: 894–899.

[68] Miller JH (1972) Experiments in molecular genetics. New York: Cold Spring Harbor Laboratory.

[69] Dombrecht B, Vanderleyden J, Michiels J (2001) Stable RK2-derived cloning vectors for the analysis of gene expression and gene function in gram-negative bacteria. Mol Plant-Microbe Interact 14: 426–4300. 11277442 10.1094/MPMI.2001.14.3.426

[70] Liu YN, Tang JL, Clarke BR, Dow JM, Daniels MJ (1990) A multipurpose broad host range cloning vector and its use to characterise an extracellular protease gene of *Xanthomonas campestris* pathovar campestris. Mol Gen Genet 220: 433–440. 2187155 10.1007/BF00391750

[71] Hirsch PR, Beringer JE (1984) A physical map of pPH1JI and pJB4JI. Plasmid 12: 139–141. 6095352 10.1016/0147-619x(84)90059-3

